# Taxonomic review of the *bifenestratus* species group of the genus *Fulvius* Stål with descriptions of two new species (Hemiptera, Heteroptera, Miridae, Cylapinae)

**DOI:** 10.3897/zookeys.796.21293

**Published:** 2018-11-15

**Authors:** Andrzej Wolski, Jacek Gorczyca, Tomohide Yasunaga, Zdeněk indra, Aleksander Herczek

**Affiliations:** 1 Institute of Biology, Opole University, Oleska 22, 45-052 Opole, Poland Opole University Opole Poland; 2 Department of Zoology, University of Silesia, Bankowa 9, 40-007 Katowice, Poland University of Silesia Katowice Poland; 3 Research Associate, Division of Invertebrate Zoology, American Museum of Natural History, New York c/o Nameshi 2-33-2, Nagasaki 852-8061, Japan American Museum of Natural History New York United States of America; 4 Department of Plant Protection, Faculty of Agrobiology, Food and Natural Resources, Czech University of Agriculture, CZ-165 21 Praha 6-Suchdol, Czech Republic Czech University of Agriculture Prague Czech Republic

**Keywords:** Australian Region, diagnosis, *
Fulvius
*, key, new species, Oriental Region, taxonomy

## Abstract

Two new species of the genus *Fulvius* Stål are described from the Philippines and Papua New Guinea. A taxonomic review of representatives of the *F.bifenestratus* species group, illustrations of the male genitalia, a color habitus image of each species, and a key to species of the group are provided.

## Introduction

*Fulvius* Stål, 1862 is a morphologically diverse, speciose genus, with more than 80 valid species worldwide; most are found in the tropical and subtropical regions (Gorczyca 2006; Schuh 2002–2013). This genus is assumed to be paraphyletic and to lack consistent diagnostic characters. A combination of characters presented by previous authors (e.g., Carvalho and Costa 1994, Gorczyca 2000, Yasunaga 2000) in diagnosing *Fulvius* (e.g., elongate, parallel-sided, rather small body; porrect head; acarinate vertex; trapezoidal pronotum; two-segmented tarsus) is also shared by many other fulviine genera. Gorczyca (2002) suggested that the Old World members of the genus *Fulvius* exhibit some interspecific variation and can be divided into the *anthocoroides* and *bifenestratus* species groups. Based on an analysis of morphological characters, Sadowska-Woda (2005) and Gorczyca (2006) proposed three species groups for a more adequate classification of the genus *Fulvius*. The *anthocoroides*, *bifenestratus*, and *bisbistillatus* (occurring in the New World) groups were supported by preliminary DNA sequencing data from only a few congeners (Sadowska-Woda et al. 2008).

Our paper provides a taxonomic review of the species belonging to the *bifenestratus* group, including diagnoses and color habitus images for all species treated. Two new species, *F.henryi* and *F.tumidipennis*, are described. Scanning electron micrographs showing selected structures of *F.bifenestratus* Poppius, *F.subnitens* Poppius, and *F.tumidipennis* sp. n. and a key to species of the *bifenestratus* group are provided.

## Materials and methods

Observations were made using an Olympus SZX12 stereomicroscope and an Olympus BX50 optical microscope. Digital images of live individuals were taken by TY using a Canon EOS Kiss digital camera body + Olympus OM-System. Scanning electron micrographs were taken using Hitachi S-3400N and Hitachi S3000N scanning electron microscopes. Measurements were taken using an eyepiece (ocular) micrometer; all measurements are given in millimeters. The structures measured were defined by Wolski (2015). Dissections of male genitalia were performed using the technique of Kerzhner and Konstantinov (1999). Terminology of the male genitalic structures follows Konstantinov (2003) for elements of the genital capsule and parameres, and Cassis (2008) in using the term “endosoma” for the male intromittent organ. The specimens examined are deposited in the institutions or personal collections listed below, with the following abbreviations:

**NHMUK** Natural History Museum, London, England

**BPBM** Department of Natural Sciences Collection, Bernice P. Bishop Museum, Honolulu, Hawaii, USA

**ISNB** Institut Royal des Sciences Naturelles de Belgique, Brussels, Belgium

**MCSN** Museo Civico di Storia Naturale, Genova, Italy

**NHMW** Naturhistorisches Museum Wien, Vienna, Austria

**NMPC** National Museum, Prague, Czech Republic

**TYCN** T. Yasunaga Collection; Nagasaki, Japan

**TLI** Tiroler Landesmuseum, Innsbruck, Austria

**US** Department of Zoology, University of Silesia, Katowice, Poland

**ZJPC** Zdeněk Jindra collection, Praha, Czech Republic

**ZMUC** Zoological Institute, University Copenhagen, Denmark

## Taxonomy

### Fulvius bifenestratus

Taxon classificationAnimaliaHemipteraMiridae

group

#### Diagnosis.

Dorsum shiny, covered with irregular, simple setae (Figs 1–12, 27–30, 37, 38); second tarsomere typically subdivided medially, without subapical claw (Figs 32, 33); aperture of pygophore subapical, oriented laterally, dorsal wall short (Figure 34); parameres strongly asymmetrical, right paramere vestigial and left paramere variable in shape (Figs 14, 15, 17, 18, 20, 22, 23, 25); endosoma membranous, without sclerites (Figs 16, 19, 21, 24, 26).

#### Discussion.

**Figures 1–9.**
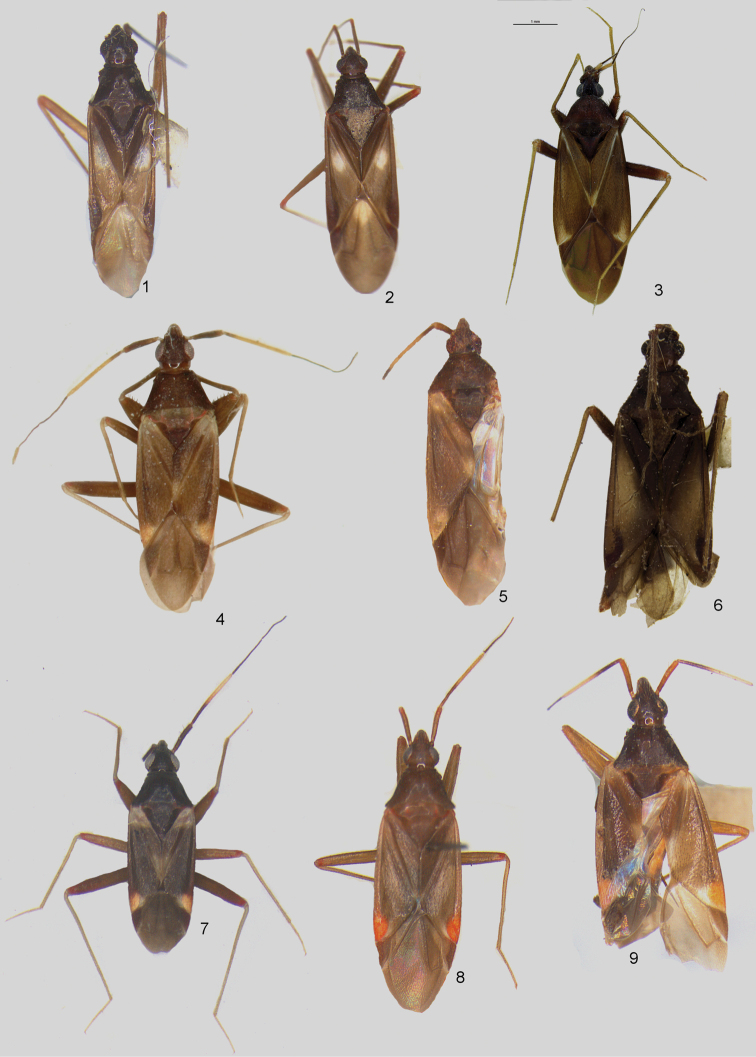
Dorsal habitus photographs of *bifenestratus* group of species of the genus *Fulvius*: **1***F.bifenestratus* (♂) **2***F.bimaculatus* (♀) **3***F.constanti* (paratype) **4, 5***F.flavicornis* (**4** ♂ **5** holotype); **6***F.henryi* (paratype, ♀) **7***F.subnitens* (♂) **8***F.thailandicus* (holotype) **9***F.tumidipennis* (paratype, ♂).

Each species of the *bifenestratus* group can be distinguished from other Old World species of *Fulvius*, members of the *anthocoroides* group, by several characters. In the *bifenestratus* group, the dorsum is shiny and covered with irregularly distributed, simple setae (Figs 1–9, 12, 29, 27, 30, 37, 38), whereas in the *anthocoroides* group the dorsum is matte, and covered with uniformly distributed, scale-like vestiture (Figs 41–44). The second tarsomere in species of the *bifenestratus* group typically is subdivided medially and the pretarsal claw lacks the subapical tooth (Figs 32, 33; Sadowska-Woda et al. 2008). In most representatives of the *anthocoroides* group the second tarsomere is not subdivided medially and the subapical tooth is present (Figs 45, 46; Sadowska-Woda et al. 2008). The aperture of the pygophore in the *bifenestratus* group is subapical and oriented laterally, with the dorsal wall relatively short (Figure 34; Sadowska-Woda et al. 2008), whereas in representatives of the *anthocoroides* group the aperture of the pygophore is oriented posteriorly and the dorsal wall is long (Figs 47, 48; Sadowska-Woda et al. 2008). The parameres in species of the *bifenestratus* group are strongly asymmetrical, with the right paramere vestigial and the left paramere quite variable in shape (Figs 14, 15, 17, 18, 20, 22, 23, 25). In contrast, members of the *anthocoroides* group have the parameres similar in size. The shape of both parameres is rather symmetrical, with the right paramere bearing a short and sharply pointed apical process and the apex of the inner surface of the paramere body possessing a short spine, whereas the left paramere is long and thin, with an incision subapically (Carvalho and Lorenzato 1978: figs 56, 57, 68, 69; Gorczyca 2002: figs 1–4; Pluot-Sigwalt and Chérot 2013: 4B, C; Yasunaga 2000: 23, 24, 28, 29; Sadowska-Woda and Gorczyca 2005: 2, 3; Yasunaga and Wolski 2017: 3A, B). The endosoma in the *bifenestratus* group is always broadly membranous and the sclerotized portion of the seminal duct is short and variable in shape (Figs 16, 19, 21, 24, 26; Sadowska-Woda et al. 2008). In species of the *anthocoroides* group, by contrast, the endosoma has sclerites or sclerotized appendages, and the sclerotized portion of the seminal duct is well developed, long and tubular (Carvalho and Lorenzato 1978: fig. 55; Pluot-Sigwalt and Chérot 2013: fig. 4A; Yasunaga 2000: 30; Yasunaga and Wolski 2017: fig. 3C; Sadowska-Woda et al. 2008). The membranous structure between the second valvulae is always absent in the *bifenestratus* group, and always present in species of the *anthocoroides* group (Sadowska-Woda et al. 2008).

Members of the *bifenestratus* group are most similar to species of the New World *bisbistillatus* group in sharing characters such as the shiny dorsum, covered with simple setae (Figs 1–9, 12, 27, 30, 37, 38, 50); the divided second tarsomere; the pretarsal claw without subapical tooth (Figs 32, 33, 52, 53); the male pygophore with aperture subapical, oriented laterally and with dorsal wall relatively short (Figs 34, 54); the parameres strongly asymmetrical with the right paramere vestigial (Figs 14, 15, 17, 18, 20, 22, 23, 25; Carvalho and Costa 1994: figs 4–6, 12–14, 19–21, 33–35, 40–42); and the membranous structure between the second valvulae absent (Sadowska-Woda et al. 2008). Representatives of the *bifenestratus* group can be distinguished from members of the *bisbistillatus* group by having the cuneus uniformly brown to dark brown (Figs 1–9), whereas in *bisbistillatus* the cuneus always possesses a pale, whitish or yellow patch at the base (Carvalho and Costa 1994: figs 1, 8, 16, 30, 37, 44). The apex of the first and second valvulae is slightly rounded or straight in the *bifenestratus* group, whereas in species of the *bisbistillatus* group the apices of the first and second valvulae are always triangular (Sadowska-Woda et al. 2008).

### Key to species of the *bifenestratus* group

**Table:** 

1	Eyes distinctly removed from pronotal collar (Figs 1–3, 10, 39); hemelytron without pale patch above cuneus (Figs 1–3, 39), covered with sparse, short setae; apical process of left paramere not elongated, distinctly broadened apically (Figs 15, 18; Carvalho and Lorenzato 1978: fig. 48)	**2**
–	Eyes only somewhat removed from pronotal collar (Figs 4–8, 27, 28, 40); hemelytron with pale patch above cuneus (Figs 4–8, 39), covered with relatively dense and long setae (Fig. 30); apical process of left paramere thin, elongated (Figs 20, 23, 25)	**4**
2	Corium and membrane without pale patches (Fig. 3)	***F.henryi* , sp. n. (Australian)**
–	Corium and membrane each with pale patches medially (Figs 1, 2)	**3**
3	Antennal segment II uniformly dark brown; apical process of left paramere bifurcated (Carvalho and Lorenzato 1978: fig. 49)	***F.bimaculatus* Poppius (Australian)**
–	Antennal segment II with whitish annulation apically; apical process of left paramere not bifurcated (Fig. 15)	***F.bifenestratus* Poppius (Oriental)**
4	Hemelytron with pale patch basally (Figs 5–7, 40); pale patch above cuneus broad (Figs 5–7, 40)	**5**
–	Hemelytron without pale patch basally; pale patch above cuneus narrow (Figs 4, 8)	**7**
5	Antennal segment II almost entirely pale yellow, narrowly darkened basally (Figs 5, 6)	***F.flavicornis* Poppius (Oriental)**
–	Antennal segment II dark brown with more or less developed annulation apically (Figs 7, 8)	**6**
6	Body length not more than 3.5 mm; apical portion of left paramere lacking subapical process ventrally (Fig. 22); endosoma not distinctly inflated (Fig. 24)	***F.subnitens* Poppius (Afrotropical, Australian, Oriental)**
–	Body length more than 3. 5 mm; apical portion of left paramere with distinct subapical process ventrally (Fig. 25); endosoma distinctly inflated (Fig. 26)	***F.tumidipennis* sp. n. (Oriental)**
7	Clavus entirely dark brown (Fig. 8)	***F.thailandicus* Gorczyca (Oriental)**
–	Clavus with thin, yellow stripe along outer margin (Fig. 4)	***F.constanti* Gorczyca (Australian)**

### Fulvius bifenestratus

Taxon classificationAnimaliaHemipteraMiridae

Poppius, 1909

Figs 1 , 10–13 , 14–16 , 39

Fulvius bifenestratus Poppius, 1909: 30, 35, 44; Bergroth 1920: 75; Carvalho 1957: 15, 1980: 643; Schuh 1995: 26; Gorczyca 2002: 18, Figs 9, 12; Sadowska-Woda and Gorczyca 2003: 336; Sadowska-Woda 2005: 20, 27, 55, 93, 105, 170, 171, 172, tab. 1, Fig. 6, tab. 15A, Figs 1–5, tab. 15B, Fig. 1, 2006a: 40; Sadowska-Woda et al. 2006: 618, 625, 632–633, Figs 6–8, 17.

#### Diagnosis.

**Figures 10–13.**
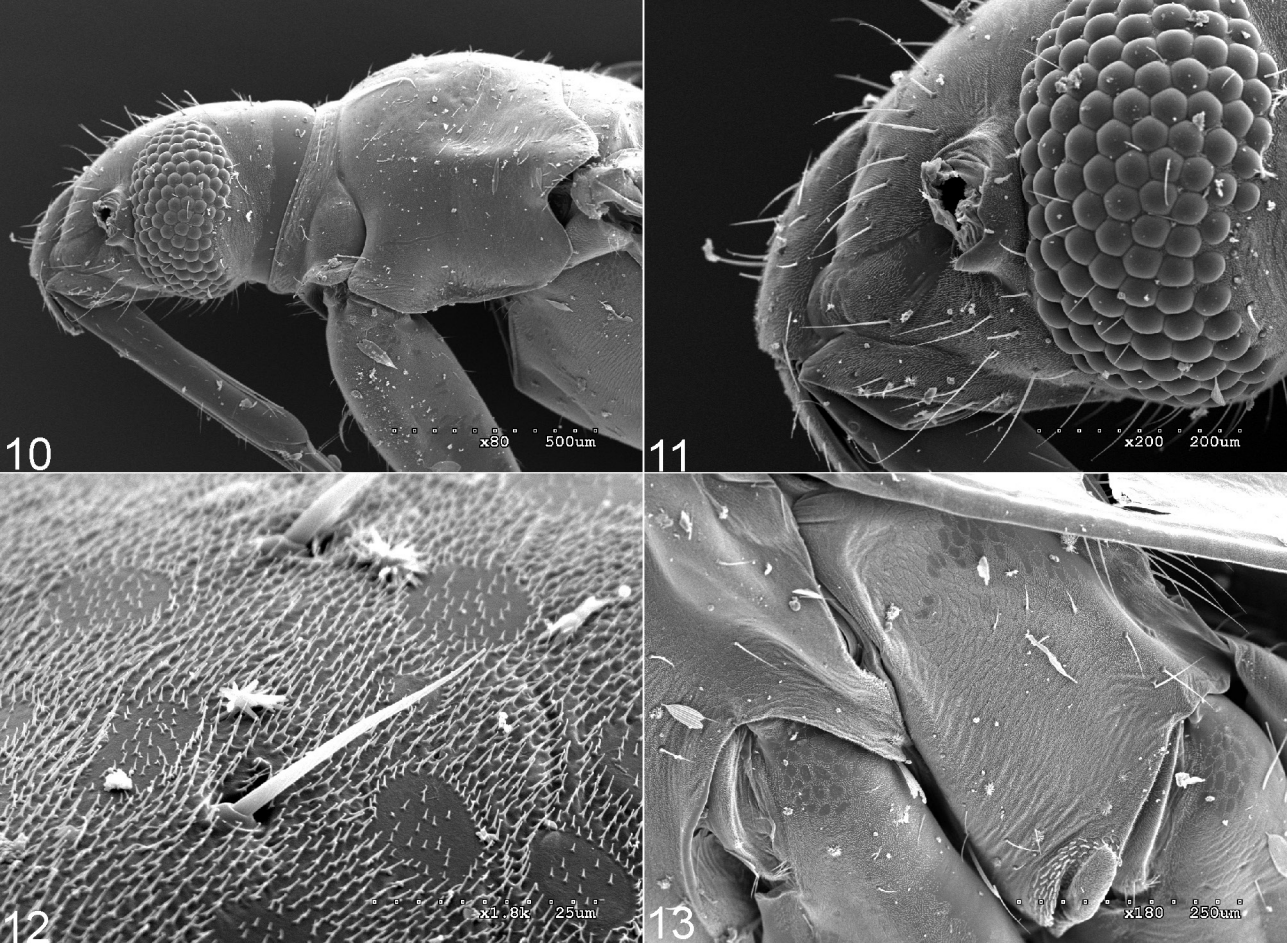
Scanning electron micrographs of *Fulviusbifenestratus*. **10** Head and thoracic pleura (left lateral view) **11** Head (left lateral view) **12** Texture and vestiture of pronotum **13** Thoracic pleura.

Eyes removed from pronotal collar (Figs 2, 10, 39); antennal segment I longer than width of head; segment II with yellow annulation apically (Figure 39); corium with distinct pale patch near base (Figs 1, 39); membrane with distinct pale patch basally (Figs 1, 39); body of left paramere thin, inner margin curved and outer margin weakly sinuate, apical process short and broadened, ventral part elongated (viewed laterally from left) (Figure 15); female genitalia as in Sadowska-Woda et al. (2006: figs 6–8, 17).

#### Remarks.

*Fulviusbifenestratus* is most similar to *F.bimaculatus* in sharing the dark brown to black corium with a large yellow patch near the base and the membrane with a yellow patch basally (Figs 1, 2). It can be distinguished by its smaller size, the coloration of antennal segment II, and form of the male genitalia. With *F.bimaculatus* and *F.henryi* it also shares the eyes removed from the pronotal collar (Figs 1–3, 10, 39); corium covered with sparse, short setae, without any pale patch over cuneus (Figs 1–3, 39); and the short and broadened apical process of the left paramere (Figs 15, 18; Carvalho and Lorenzato 1978: fig. 49), but is distinguished by the shape of the male genitalia.

#### Biology.

Unknown.

#### Distribution.

Brunei (Temburong), Indonesia (Mentawei Isl., Sulawesi), Malaysia (Borneo: Sabah), Philippines (Mindanao: Misamis Oriental Province), Thailand (Nakhon Ratchasima).

#### Type material.

**Holotype** ♀: INDONESIA: Mentawei, Sipora, Sereinu V-VI, 94 [1894], Modigliani; Museo Civ. Genova; *Fulviusbifenestratus* n. sp., B. Poppius det. (MCSN).

#### Additional material examined.

7 ♀♀ and 4 ♂♂: MALAYSIA: Sabah, British N. Borneo, Tawau, Quoin Hill, Cocoa Res. Sta., 5. IX. 1962; Y. Hirashima, Light Trap, Bishop; 2 ♀♀: British N. Borneo, Tawau, Quoin Hill, 3–7. VII. 1962; H. Holtmann, Light Trap, Bishop; ♂: Same data, except collected in jungle, H. Holtmann, Light Trap, Bishop; ♂ and ♀: British N. Borneo, Tawau, Quoin Hill, 15–20. VII. 1962; H. Holtmann, Light Trap, Bishop; ♀: British N. Borneo, Tawau, Quoin Hill, 8–14. VII. 1962; H. Holtmann, Light Trap, Bishop; ♀: North Borneo (SE), Forest Camp, 19 km, N. of Kalabakan, 60 m, 18. X. 1962; K. J. Kuncheria Collector, Bishop; ♀: North Borneo (SE), Forest Camp, 19 km, N. of Kalabakan, 60 m, 18. X. 1962; Y. Hirashima Collector, Bishop; ♂: North Borneo (SE), Tawau, Quoin Hill, Cocoa Res. Sta., 13. IX. 1962; Y. Hirashima, Malaise Trap, Bishop; ♂: British N. Borneo, Tawau, Quoin 11;ill, Cocoa Res. Sta., 24. IX. 1962; Y. Hirashima, Light Trap, Bishop; ♂: British N. Borneo, Tawau, Quoin Hill, Cocoa Res. Sta., 4. IX. 1962; Y. Hirashima, Light Trap, Bishop; ♂: British N. Borneo, Tawau, Quoin Hill, Cocoa Res. Sta., 3. IX. 1962; Y. Hirashima, Light Trap, Bishop; ♀: P. I., Misamis OR., Mt. Balatukan, 10 km SW of Gingoog, 1000–2000m, 1–5. V. 1960; H. Torrevillas Collector. (3 ♂♂ and 4 ♀♀ in US, rest in BPBM); ♀: Light Trap; Sarawak: foot of Mt. Dulit, Junction of rivers, Trnjar & Lejok, 29. viii. 1932; Oxford Univ. Exp., B.M. Hobby, A.W. Moore, B. M. 1933-254; ♂: 125W. v. light; BRUNEI: Temburong District, ridge NE of Kuala Belalong, approx. 300 m alt., October 1992, J H Martin coll., B M 1992 – 172; Fulviini, det. G. Stonedahl, 19; ♀: Rothamsted light trap, site 1, 200m., H. Barlow; Indonesia: Sulawesi Utara, Dumoga-Bone N. P., February, 1985; ♀: at light; INDONESIA: SULAWESI UTARA Dumoga-Bone N. P., April, 1985; R. Ent. Soc. Lond, PROJECT WALLANCE, B. M., 1985 – 10, Clarck’s Camp 1140 m; J.H. Martin Coll. (NHMUK); ♀: THAILAND: Nakhon Ratchasima Sakaerat Environmental Research Station, 14°30'N, 101°55'E, 400 m, light trap, 16 Sep 2008, T. Yasunaga; ♀: same data except for date 15 Sep 200; ♀: THAI: Nak. Ratchasima Sakaerat Forest R.S., 14°30'N, 101°55'E, 400 m, LT, 31 August 2008, T. Yasunaga; ♀: THAI: Nk. Ratchasima Sakaerat Environ. R. S., 14°30'N, 101°55'E, 400 m, LT 12–14.vi.2009, Yasunaga & Yamada (TYCN).

### Fulvius bimaculatus

Taxon classificationAnimaliaHemipteraMiridae

Poppius, 1909

Figure 2

Fulvius bimaculatus Poppius, 1909: 30, 36, 44; Bergroth 1920: 75; Carvalho 1957: 15, 1980: 643; Carvalho and Lorenzato 1978: 135, Figs 46–49, 51; Schuh 1995: 26; Gorczyca 2002: 18, 21, Fig. 10; Sadowska-Woda and Gorczyca 2003: 336; Sadowska-Woda 2005: 20, 25, 27, 55, 93, 106, 170, 171, 172, Tab. l, Fig. 3, Tab. 12, Fig. l, Tab. 16, Figs 1–5; Gorczyca 2006: 41.

#### Diagnosis.

Eyes removed from pronotal collar (Figure 2); antennal segment II entirely dark brown; corium dark brown except for large, yellow patch near base (Figure 2); membrane with large, yellow patch basally (Figure 2); apical process of left paramere short and broadened, bifurcated (Carvalho and Lorenzato 1978: Figure 49).

#### Remarks.

*Fulviusbimaculatus* is most similar to *F.bifenestratus* (see diagnosis of *F.bifenestratus*), but it can be distinguished by the body size, the coloration of antennal segment II, and the male genitalia. With *F.bifenestratus* and *F.henryi* it also shares the similar head shape, corial coloration and vestiture, and a short and broadened left paramere apical process, but it can be distinguished by the overall structure of the male genitalia.

#### Biology.

Unknown.

#### Distribution.

Papua New Guinea.

#### Type material.

**Lectotype** (♂, designated by Carvalho and Lorenzato 1978): PAPUA NEW GUINEA: N. Guinea S. E., Moroka, 1300 m, LORIA VII-30 93; Museo Civ. Genova; paralectotype ♂: N. Guinea S. E., Moroka, 1300 m, LORIA VII-30 93; Museo Civ. Genova (MCSN).

#### Additional material examined.

2 ♀♀ and ♂: PAPUA NEW GUINEA: Kokoda, 1200ft., ix 1933, L. E. Cheesman, B. M. 1934-321; ♀: Papua: Kokoda, 1200ft., viii-ix 1933, L. E. Cheesman, B. M. 1933-427; ♂: Papua: Kokoda, 1200 ft., viii 1933, L. E. Cheesman, B. M. 1933-427; ♀: Sten. No. 137; New Guinea: Morbe Dist., Herzog Mts., Vagau, C. 4 000 ft., 4–17. I. 1965; M. E. Bachus, B. M. 1965-120; ♂ and 2 ♀♀: W. New Guinea: Mt. Nomo. S. of Mt. Bougainville, 700 ft., ii. 1936; ♀: N. Dutch New Guinea: Waigeu. Camp 1., Mt. Nok., 2,500 ft., v. 1938, L. E. Cheesman., B. M. 1938-593; ♂: Fluorescent Mv Trap; Papua New Guinea: Morobe Prov. coast, Buso, 30. ix 1979, J. H. Martin coll.; Brit. Mus., 1980-150; ♀ and ♂: Dutch New Guinea: Humboldt Bay Dist., Bewani Mts., 400 metres, vii. 1937; W Stüber, B. M, 1938-177; 2 ♀♀: Dutch New Guinea: Cyclops Mts. Sabron. Camp: 2, 000 ft., vi. 1936, L. E. Cheesman, B. M. 1936-271; ♀: Dutch New Guinea, Mt. Cyclops, 4,000 ft. 12. iii. 1936, L. E. Cheesman, B. M., 1936-271; ♀: Dutch New Guinea, Cyclops Mts., Sabron, Camp I: 1,200 ft. 22. v. 1936, L. E. Cheesman, B. M., 1936-271; ♂: Dutch New Guinea, Cyclops Mts., Sabron, Camp I: 1,200 ft. 15. v. 1936, L. E. Cheesman, B. M., 1936-271; Dutch New Guinea, Cyclops Mts., Sabron, Camp 2: 2,000 ft.. v. 1936, L. E. Cheesman, B. M., 1936-271; ♀: Dutch New Guinea, Mt. Cyclops, Camp I: 3,500 ft. iii. 1936, L. E. Cheesman, B. M., 1936-271 (5 specimens in US, remainder in BMNH); ♂: PNG: New Guinea: NE, Morobe Prov.: Mt. Missim, S side, 2000 m, 15. VI. 1984; permethrin log of *Castanopsisacuminatissima*, mature canopy; W. C. Gagne & Urep session III, Colis sample #8, tree #330B; ♀: PNG: New Guinea: NE: Madang Prov.: Baku Forest Stn., 80 m, 4–12. II. 1978; At light; W. C. Gagne Coll, Bishop Museum, Acc. #1980, 4; 2 ♂♂: New Guinea: NE: Morobe Distr.: Kabwun to Ilaka, 4. VIII. 1966; G. A. Samuelson Collector, Bishop Museum; ♀: New Guinea: NE, Wau, 1200m, 30–31. x. 1964; M. V. Lamp, J. Sedlacek, Coll., Bishop Museum; ♂: New Guinea, Morobe Distr., Wau, 15. VIII. 1972, G. G. E. Scudder; MV Light Trap, G. G. E. Scudder; Bishop Museum Coll., Acc. 1981-522; ♀: New Guinea: NE, Wau, Morobe Distr., Mt. Missim, 1600 m, 1. V. 1974; Light Trap; Thane Pratt collector, Wau Ecology Inst. (Bishop); Bishop Museum, Accession 1980, 4; ♀: PNG: New Guinea: (NE), W. Sepik Prov., Feramin, 1500 m, 2. VII. 1976; collected at light; W. C. Gagne coll., Bishop Museum (two specimens in US, remainder in BPBM).

### Fulvius constanti

Taxon classificationAnimaliaHemipteraMiridae

Gorczyca, 2004

Figure 3

Fulvius constanti Gorczyca, 2004: 154, figs 1–3; Gorczyca 2006: 41; Sadowska-Woda 2005: 21, 26, 27, 57, 93, 106, 170, 171, 172, tab. 19 figs 1–3; Sadowska-Woda et al. 2006: 619, 625, 633, 634, Figs 2, 21.
Fulvius nigricornis : Carvalho and Lorenzato 1978: 136, figs 50–53, 88, (nec Poppius 1909).

#### Diagnosis.

Eyes contiguous with pronotal collar (Figure 3); antennal segment II dark brown with broad, yellow annulation apically (Figure 3); corium with pale patch over cuneus (Figure 3); clavus with pale, thin stripe along outer margin and small, yellow patch apically (Figure 3); male genitalia as in Gorczyca (2004: figs 1–3).

#### Remarks.

*Fulviusconstanti* is most similar to *F.flavicornis*, *F.subnitens*, *F.thailandicus*, and *F.tumidipennis* in having the eyes contiguous with the pronotal collar; the corium covered with dense setae, a pale patch over the cuneus; and the left paramere with an elongate apical process. It can be distinguished, however, by the absence of pale patches basally on the corium; the clavus with a thin, yellow stripe along its outer margin; and the shape of the male genitalia.

#### Biology.

Unknown.

#### Distribution.

Papua New Guinea.

#### Type material.

**Holotype** ♂: Coll. I. R. Sc. N. B., Canopy Mission Papua New Guinea (Madang prov.): Batiteta, 08. VI. 1993, Light trap M1. Leg. Olivier Missa (ISNB); paratypes: ♀ and ♂: same data as holotype; ♀: Coll. I. R. Sc. N. B., Canopy Mission Papua New Guinea (Madang prov.): Batiteta, 03. VI. 1996, Light trap M7. Leg. Olivier Missa; ♂: Coll. I. R. Sc. N. B., Canopy Mission Papua New Guinea (Madang prov.): Batiteta, 13. IV. 1996, Light trap AR16. Leg. Olivier Missa (ISNB); ♂: Coll. I. R. Sc. N. B., Canopy Mission Papua New Guinea (Madang prov.): Batiteta,19. VI. 1996, Light trap AR22. Leg. Olivier Missa; ♂: Canopy Mission Papua New Guinea (Madang prov.): Batiteta, 02. VII. 1996, Light trap AR60. Leg. Olivier Missa, Coll. I. R. Sc. N. B (US).

### Fulvius flavicornis

Taxon classificationAnimaliaHemipteraMiridae

Poppius, 1909

Figs 4 , 5 , 6 , 20 , 21

Fulvius flavicornis Poppius, 1909: 30, 34, 44; Bergroth 1920: 76; Carvalho 1957: 17, 1980: 644; Schuh 1995: 27; Sadowska-Woda 2005: 102; Gorczyca 2006: 35.

#### Diagnosis.

Eyes contiguous with pronotal collar (Figs 4, 5); antennal segment II yellow, except basal one sixth brownish (Figs 4, 5); basal and apical portions of corium and apex of clavus yellow (Figs 4, 5); left paramere with apical process and paramere body forming obtuse angle, paramere body strongly narrowed medially, broadened apically, apical process elongated, broadened basally, weakly tapering toward apex (Figure 20).

#### Remarks.

*Fulviusflavicornis* is most similar to *F.subnitens* and *F.tumidipennis* in sharing a yellow patch on the base and apex of the corium and apex of clavus, and the left paramere apical process elongate. *Fulviusflavicornis* can be distinguished by the coloration of antennal segment II and by the male genitalia.

#### Redescription.

**Female**. **Coloration** (Figs 4, 5). Dorsum dark brown, with yellowish areas. ***Head***. Dark brown; antennal segment I brownish basally then dark yellowish; segment II yellow, with narrow, brown annulation basally; segments III and IV dark brown; rostrum yellow brown. ***Thorax***. *Pronotum*. Dark brown. *Thoracic pleura*. Dark gray brown. *Hemelytron*. Dark fuscous, embolium, corium, and clavus yellow basally and apically; cuneus dark brown; membrane dark gray. *Legs*. Forecoxae yellow at basal half, then brownish; remaining segments of each leg missing. ***Abdomen***. Brownish tinged with indistinct, yellow patches. **Structure and vestiture** (Figs 4, 5). Dorsal surface covered with relatively dense and long setae. ***Head***. Eyes contiguous with pronotal collar.

**Male**. Similar to ♀ in coloration, texture, and vestiture. ***Thorax***. *Legs*. Tibiae, femora and tarsi dark brown with dirty yellowish areas. **Male genitalia** (Figs 20, 21). *Left paramere* (Figure 20). Apical process broadened on basal one third, thin, tapering toward apex on apical two thirds; paramere body broadened basally and apically, narrowed medially. *Aedeagus* (Figure 21). Membranous, secondary gonopore undifferentiated.

#### Measurements.

♀/♂ (holotype measurements first). *Body*. Length 4.4/3.7, width 1.5/1.1. *Head*. Length of head 0.6/0.5, width 0.5/0.5, interocular distance 0.24. *Antenna*. Length of segment I 0.5/0.5, II 1.2/0.9, III 0.6 (♂, missing in ♀), IV 0.7 (♂, missing in ♀). *Labium* (♀, immeasurable in ♂). Length of segment I 0.7, II 0.9, III 1.1, IV 0.5. *Pronotum*. Length of pronotum 0.6/0.5, length of anterior margin 0.5/0.5, lateral margins 0.7/0.6, posterior margin 1.3/1.3.

#### Biology.

Unknown.

#### Distribution.

Indonesia (Sumatra: Sirambas).

#### Type material.

**Holotype** ♀: INDONESIA: SUMATRA, SI-RAMBÉ, XII.90 – III.91, E. MODIGLIANI; Museo Civ. Genova; *Fulviusflavicornis* n. sp.; HOLOTYPUS, *Fulviusflavicornis* B. Poppius, 1909 (MCSN).

#### Additional examined material.

♂: MALAYSIA: W. Perak, 40 km SE of IPOH, 900 m, Banjaran Titi Wangsa, RINGLET, 29.iii.15.iv 2004, Čechowsky Petr lgt. (NHMW).

### Fulvius henryi

Taxon classificationAnimaliaHemipteraMiridae

Wolski, Gorczyca & Yasunaga sp. n.

http://zoobank.org/13E7A938-0685-427B-9F26-02CE8680CF3B

Figs 6 , 17–19

Fulvius unicolor : Carvalho and Lorenzato 1978, nec Poppius 1909: 29, 36.

#### Diagnosis.

Eyes removed from pronotal collar (Figure 6); corium entirely dark brown, without pale areas (Figure 6); left paramere stout, paramere body strongly curved, broadened basally and apically, apical process short, broadened apically (Figure 18).

#### Remarks.

*Fulviushenryi* is most similar to *F.bifenestratus* and *F.bimaculatus* in having the corium with short and sparse setae, lacking a pale patch over the cuneus, and in having the apical process of the left paramere short and broadened (Figs 15, 18; Carvalho and Lorenzato 1978: fig. 49). It can be distinguished by lacking a large yellow patch near the base of the corium and the shape of the male genitalia.

#### Description.

**Female**. **Coloration** (Figure 6). Dorsum uniformly dark brown. ***Head***. Dark brown; antenna dark brown; labium brown to dark brown. ***Thorax***. *Pronotum*. Dark brown. *Mesoscutum and scutellum*. Dark brown. *Thoracic pleura*. Proepimeron, mesepisternum and mesepimeron brown. *Hemelytron*. Dark brown; membrane brown to dark brown, venation brown. *Legs*. Coxae brown to dark brown; femora chestnut, sometimes slightly tinged with red apically; tibiae brown, slightly paler than femora; tarsi brown to pale brown, long. ***Abdomen*.** Chestnut to dark brown. **Structure, texture, and vestiture** (Figure 6). Dorsum covered with short, fine, pale setae. ***Head***. Antennal segment II almost cylindrical, covered with dense, short setae.

**Male.** Similar to female in structure, texture, and vestiture. **Male genitalia** (Figs 17–19). *Right paramere* (Figure 17). Irregularly shaped; apical process short and thin; paramere body ovoid. *Left paramere* (Figure 18). Apical process curved and broadened basally, thin, nearly cylindrical apically, convex on dorsal surface of extreme apex; paramere body broadened on basal half, thin on apical half. *Aedeagus* (Figure 19). Endosoma membranous, thin, broadened toward apex.

#### Measurements.

♀/♂ (* holotype measurements). Body length 4.2–4.50*/3.8, width 1.4–1.5*/1.2. *Head*. Length 0.6–0.7*/0.4, width 0.6/0.5, interocular distance 0.2/0.2. *Antenna*. Length of segment I 0.6/0.6, II 1.07/1.0, III 0.7. Labium. Length of segments I 0.6, II 0.7, III and IV together 1.4. *Pronotum*. Length 0.5/0.5, width of anterior margin 0.6, length of lateral margin 0.7–0.70*, width of posterior margin 1.2–1.2*/1.0.

#### Distribution.

Papua New Guinea, including New Britain and New Ireland.

#### Etymology.

It gives us great pleasure to dedicate this new species to Dr. Thomas J. Henry on the occasion of his 70^th^ birthday and for his many outstanding contributions to the study of Heteroptera.

#### Type material.

Holotype (♀): PAPUA NEW GUINEA: New Britain, Gazelle Pen., Mt. Sinewit, 5–9. XI. 1962; J. Sedlacek, Malaise Trap, Bishop (US); paratype (♀): New Britain, Gazelle Pen., Mt. Sinewit, 10. XI. 1962; Light Trap, J. Sedlacek, Bishop; *Fulviusunicolor* Popp. det. J.C.M. Carvalho 19; paratype (♀): New Britain, Gazelle Pen., Mt. Sinewit, 5- 10. XI. 1962; J. Sedlacek Collector, Bishop; *Fulviusunicolor* Popp. Det. J.C.M. Carvalho 19; paratype (♀): New Britain, Gazelle Pen., Mt. Sinewit, 900 m, 5–9. XI. 1962; J. Sedlacek, Malaise Trap, Bishop; *Fulviusunicolor* Popp. det. J.C.M. Carvalho 19 (US); paratype (♂): Papua New Guinea: New Britain, Gazelle Pen., Mt. Sinewit, 5–9. XI. 1962; J. Sedlacek, Malaise Trap, Bishop; Carvalho to Drake coll. 1993; paratype (♂): New Britain, Gazelle Pen., Upper Warangoi, Illugi, 25-26.XI.1962; J. Sedlacek, Malaise Trap, Bishop; Carvalho to Drake coll. 1993; paratype (♀): New Irland (SW), Ridge above "Camp Bishop" 15 km up Kait R. 250-500 m, VI.12.1956; E. J. Ford, Jr. Collector; Carvalho to Drake coll. 1993; paratype (♀): New Guinea: NE, Finisterre Range, Saidor, Kiambavi Vill., VII-22-29.1958; W. W. Brandt Collector Bishop; paratype (♀): NE New Guinea: Umboi I, c8km, WNW Lab Lab., 300 m, 8-19.II.1967; G.A. Samuelson Light trap, Bishop; Carvalho to Drake Coll. 1993 (USNM).

**Figures 14–26.**
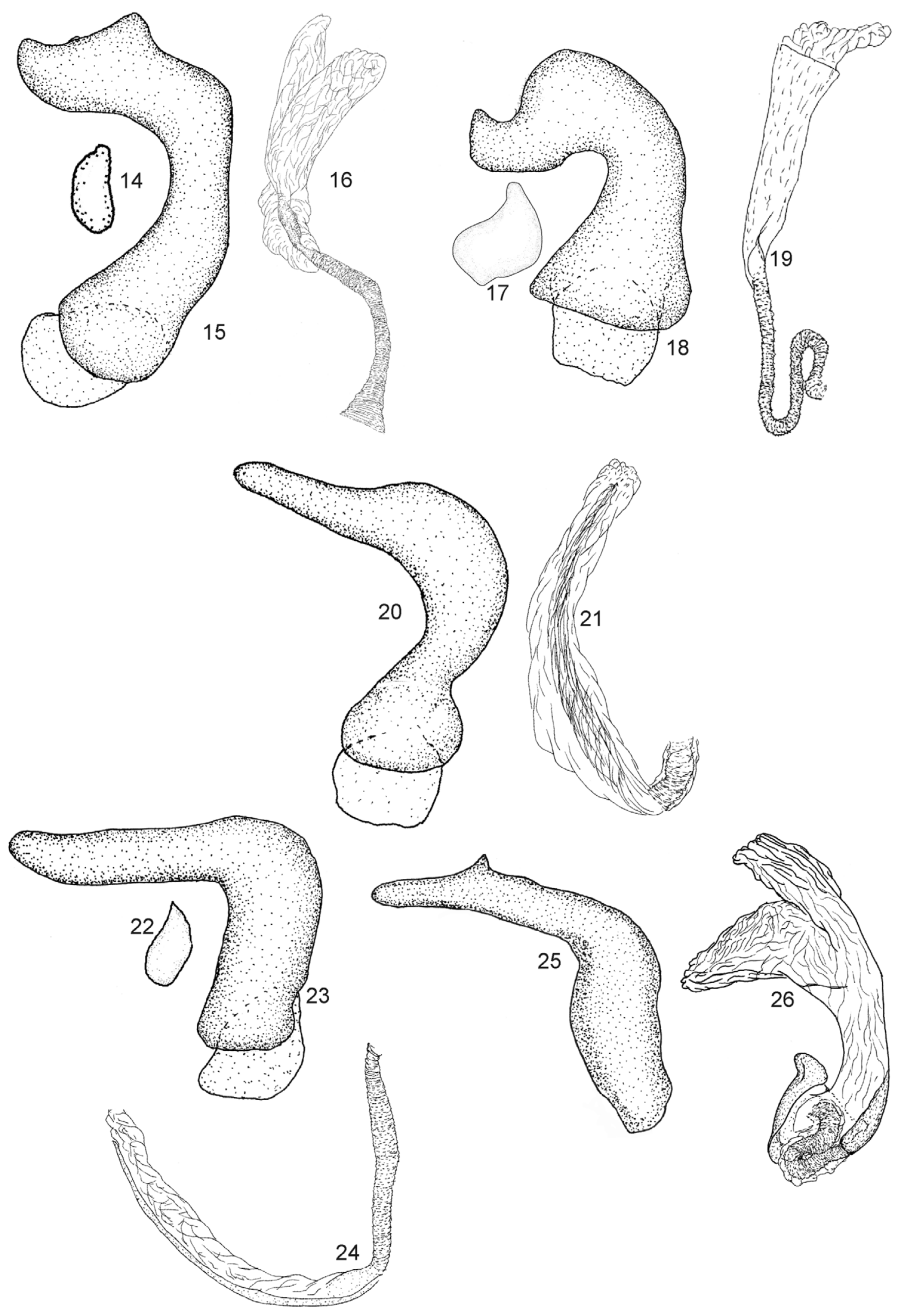
Male genitalia of *F.bifenestratus* (**14–16**), *F.henryi* (**17–19**), *F.flavicornis* (**20, 21**), *F.subnitens* (**22–24**), and *F.tumidipennis* (**25, 26**) **14, 17, 22** right paramere (dorsal view) (**15, 18, 20, 23, 25**) Left paramere (left lateral view) **(16, 19, 21, 24, 26**). Endosoma (left lateral view).

### Fulvius subnitens

Taxon classificationAnimaliaHemipteraMiridae

Poppius, 1909

Figs 7 , 27–34 , 22–24 , 40

Fulvius subnitens Poppius 1909: 30, 34, 44; Bergroth 1920: 77; Carvalho 1957: 19, 1980: 644, 1980: 652; Carvalho and Lorenzato 1978: 139, figs 58–61, 89; Schuh 1995: 29; Gorczyca 2000: 53, 65, 83, 2002: 21; 2006: 41; Sadowska-Woda and Gorczyca 2003: 336; Sadowska-Woda, 2005: 24, 29, 67, 93, 106, 170, 171, 172, tab. 3, Fig. 6, tab. 37, Figs 1–4A, B, tab. 44, Fig. 1A, B, tab. 48, Fig. 1A, B; Sadowska-Woda et al. 2008: 414, 415; Henry et al. 2011: 128, 129, 133, 134 (Figs 5, 6); Yasunaga 2017: 51 (Fig. 1).
Fulvius sauteri Poppius (synonymized by Gorczyca 2006: 41): Poppius 1915: 50; Bergroth 1920: 77; Carvalho 1957: 19, 1980: 652; Gaedike 1971: 151; Schuh 1995: 29; Kerzhner and Josifov 1999: 8; Kerzhner and Schuh 2001: 269.
Fulvius nakatai Yasunaga & Miyamoto (synonymized by Yasunaga and Wolski 2017): Yasunaga and Miyamoto 2006: 722, 731 (Fig. 5F–H); Yasunaga and Wolski 2017: 588–590 (Figs 1H, 2D).

#### Diagnosis.

Eyes contiguous with pronotal collar (Figs 7, 28, 40); antennal segment II dark brown with large, yellow annulation apically Figs 7, 28, 40); corium covered with dense setae, with distinct pale patches basally and apically (Figs 7, 28, 40); left paramere with apical process and paramere body forming right angle, paramere body and apical process nearly cylindrical, apical process elongate and weakly narrowed apically (Figure 22).

#### Remarks.

*Fulviussubnitens* is most similar to *F.flavicornis* and *F.tumidipennis* in having the corium with a pale patch basally (Figs 5, 6, 9, 40), but it is distinguished by the structures of the male genitalia.

#### Biology.

Unknown. Adults have been collected at light traps and from rotten logs.

#### Distribution.

Brunei (Temburong), Fiji, Indonesia (S. Sulawesi: Bogani Nani Wartabone National Park; Bali: Ubud), Japan (Okinawa Island), E. & W. Malaysia, Papua New Guinea, Samoa, Seychelles (Mahe I), Solomon Islands, Taiwan, Thailand (Nakhon Ratchasima), Tanzania, USA (Virginia) (Gorczyca 2006; Henry et al. 2011; Yasunaga and Wolski 2017).

#### Type material.

**Lectotype** ♂ (designated by Carvalho and Lorenzato 1978): PAPUA NEW GUINEA, Mer., Bujakori, 1890; *Fulviussubnitens* Poppius; Museum Zool. Helsingfors, type no. 9993, (ZMHU); paralectotype (?): N. Guinea, Biró; Stephansort, Astrolabe Bay; *Fulviussubnitens* n. sp., B. Poppius det; Typus (HNHM).

**Figures 27–34.**
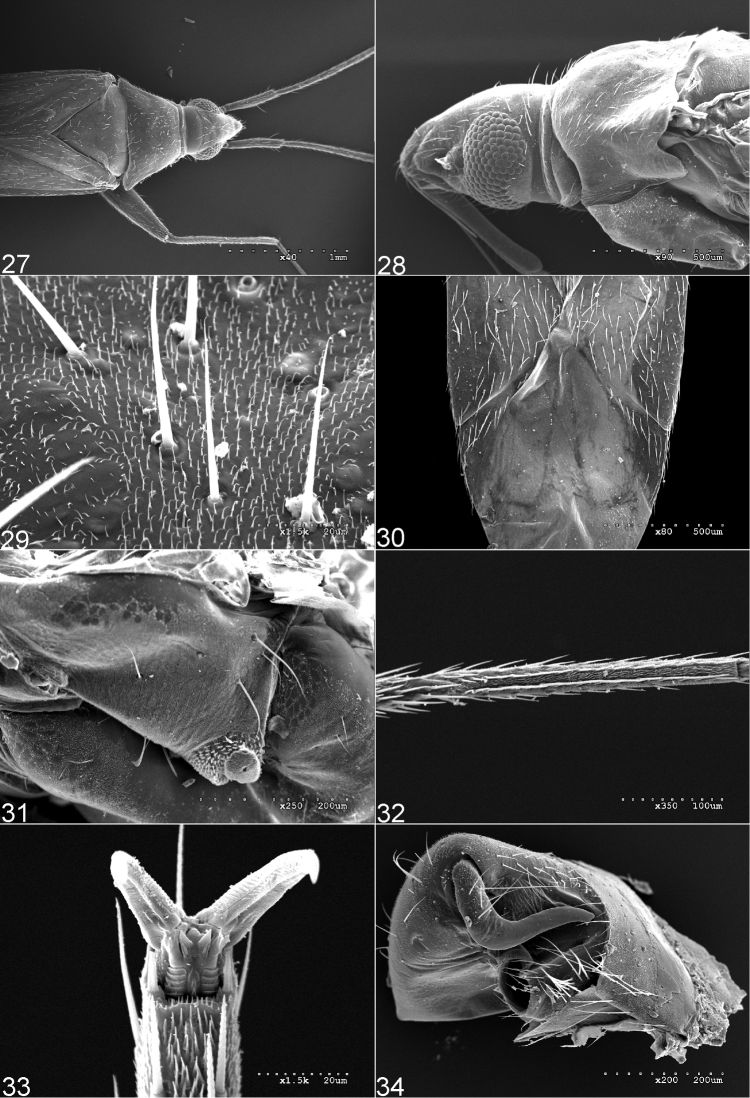
Scanning electron micrographs of *Fulviussubnitens*: **27** Dorsal view **28** Head and thoracic pleura (left lateral view) **29** Texture and vestiture of frons **30** Structure, texture, and vestiture of hemelytron **31** Metepisternum **32**. Metatarsus **33** Pretarsal structure **34** Pygophore.

#### Additional material examined.

♀ and ♂: MALAYSIA: Sarawak, Skrang River, 30 km upstream, 15 VII 92, Heiss; ♂: MALAYSIA, Pengang on *Hevea* sp. XI 84, Heiss; 8 ♀♀ and ♂: 125W. v. light; BRUNEI: Temburong District, ridge NE of Kuala Belalong, approx. 300 m alt., October 1992, J H Martin coll., B M 1992 – 172; 2 ♂♂: MALAYSIA: Kedah, Pulau Langkawi NW, Datai Rainforest, 2–10 XI 2002, E.Heiss; Sammlung-Collection Ernst Heiss Innsbruck-Austria; sp. 3; ♀: INDONESIA, Central BALI, Ubud, Maya Hotel LF, 4 XI 2005, E. HEISS; Sammlung-Collection Ernst Heiss Innsbruck-Austria (TLI); ♂: 26 XII 1994, Malays, Pahang Rov. Kulala Lipis; Dr. Wolfgang G. Ullrich collection (US); 2 ♀♀: MALAYSIA: Sarawak, Mulu NP., 3.-5. 3., 1993; leg. H. Zettel (14); (d) Benarat Inn, am Licht, 3.-5. 3 (NHMW); 5 ♂♂: at light; INDONESIA: Sulawesi Utara [= South Sulawesi], Dumoga-Bone N. P., 13 February 1985; site 8, 540 m, Tompah Transect, J. D. Holloway; R. Ent. Soc. Lond, Project Wallace, B. M., 1985 - 10; ♂: same locality, 13 February 198:?; site 8, 540 m, Tompah Transect, J. D. Holloway; R. Ent. Soc. Lond, Project Wallace, B. M., 198510; ♂: Edw. Jacobson, Gun. Teleman, Sum. 1917 all housed in NHMUK; 3 ♀♀ and ♂: at light (1 ♀: under bark); Indonesia: Sulawesi Utara, Dumoga Bone N. P., February; Sites 10 + II. 1040 m., Tumpah Transect, J. D. Holloway; R. Ent. Soc London., Project Wallace, B. M. 1985-10 (NHMUK); 3 ♀♀ and 3 ♂♂: Sungai Segama, W. side suspension bridge, 150m, 30.XI.1989; BORNEO: Sabah, DANUM VALLEY, 70 km W Lahad Datu, M.J. & J.P. Duffels; sample Sab. 49, under storey secondary growth/canopy riverine rainforest at light; ♀: Nature Trail 150 28.XI.1989; MALAYSIA: Sabah, DANUM VALLEY, 70 km W Lahad Datu, M.J. & J.P. Duffels; sample Sab. 44 open area in primary rainforest; understorey/canopy, at light; ♀: Sungai Segama, W. side suspension bridge, 150m, 10.XII.1989; MALAYSIA: Sabah, DANUM VALLEY, 70 km W Lahad Datu, M.J. & J.P. Duffels; sample Sab. 62, understorey secundary growth/canopy riverine rainforest at light; 5 ♀ and 2 ♂: MALAYSIA, Sarawak, 10–19 March 1994, Kapit. distr. Sebong env., Baleh riv., P. Bilek lgt.; EX COLLECTIO Z. JINDRA, PRAGUE (ZJPC); 1 ♀: INDONESIA, Irian Jaya, Kota Biak, 12.xii.2006, S. Bílý lgt.; COLLECTIO NATIONAL MUSEUM, Praha, Czech Republic (NMPC); ♀ and 2 ♂: 1 ♀♀: THAI: Nak. Ratchasima Sakaerat Forest R.S., 14°30'N, 101°55'E, 400 m, LT, 15 Sep 2008, T. Yasunaga; 3 ♀: THAILAND: Nakhon Ratchasima, Sakaerat Environ. R. S., 14°30'N, 101°55'E, 400 m, LT 12–14.vi.2009, Yasunaga & Yamada (TYCN).

### Fulvius thailandicus

Taxon classificationAnimaliaHemipteraMiridae

Gorczyca in Sadowska-Woda & Gorczyca, 2003

Figure 8

Fulvius thailandicus Gorczyca in Sadowska-Woda and Gorczyca 2003: 336, figs l-5; Sadowska-Woda 2005: 24, 26, 93, 106, tab., 8, Fig. 5, tab. 9, Fig. 6, tab. 12, Fig. 3A, B, tab. 13, Fig. 3A, B, tab. 48, Fig. 2A, B; Gorczyca 2006: 42.

#### Diagnosis.

Eyes contiguous with pronotal collar (Figure 8); antennal segment II dark brown with broad, yellow annulation apically (Figure 8); corium covered with dense setae, with yellow-orange patch above cuneus (Figure 8); male genitalia as in Sadowska-Woda and Gorczyca (2003: figs 2–4).

#### Remarks.

*Fulviusthailandicus* is most similar to *F.constanti*, *F.flavicornis*, *F.subnitens*, and *F.tumidipennis* in having the eyes contiguous with the pronotal collar (Figs 5–7, 9), the corium covered with dense setae and a pale patch above the cuneus (Figs 5–7, 9), and the apical process of the left paramere elongate (Figs 20, 22, 25). *Fulviusthailandicus* can be distinguished by the orange patch above the cuneus and structures of the male genitalia.

#### Biology.

Unknown.

#### Distribution.

Thailand (Chiang Mai Province).

#### Type material.

**Holotype** ♂: THAILAND, Doi Suthep – DoiPui natn. Park, Doi Pui road, 1000 m, 23–26. x. 1979, Zool. Mus. Copenhagen Exped.; **paratype** ♂: the same data as holotype (ZMUC).

### Fulvius tumidipennis

Taxon classificationAnimaliaHemipteraMiridae

Wolski, Gorczyca & Yasunaga sp. n.

http://zoobank.org/5F18A418-B185-4BF2-84FC-8BC75781B6F9

**Figures 35–38.**
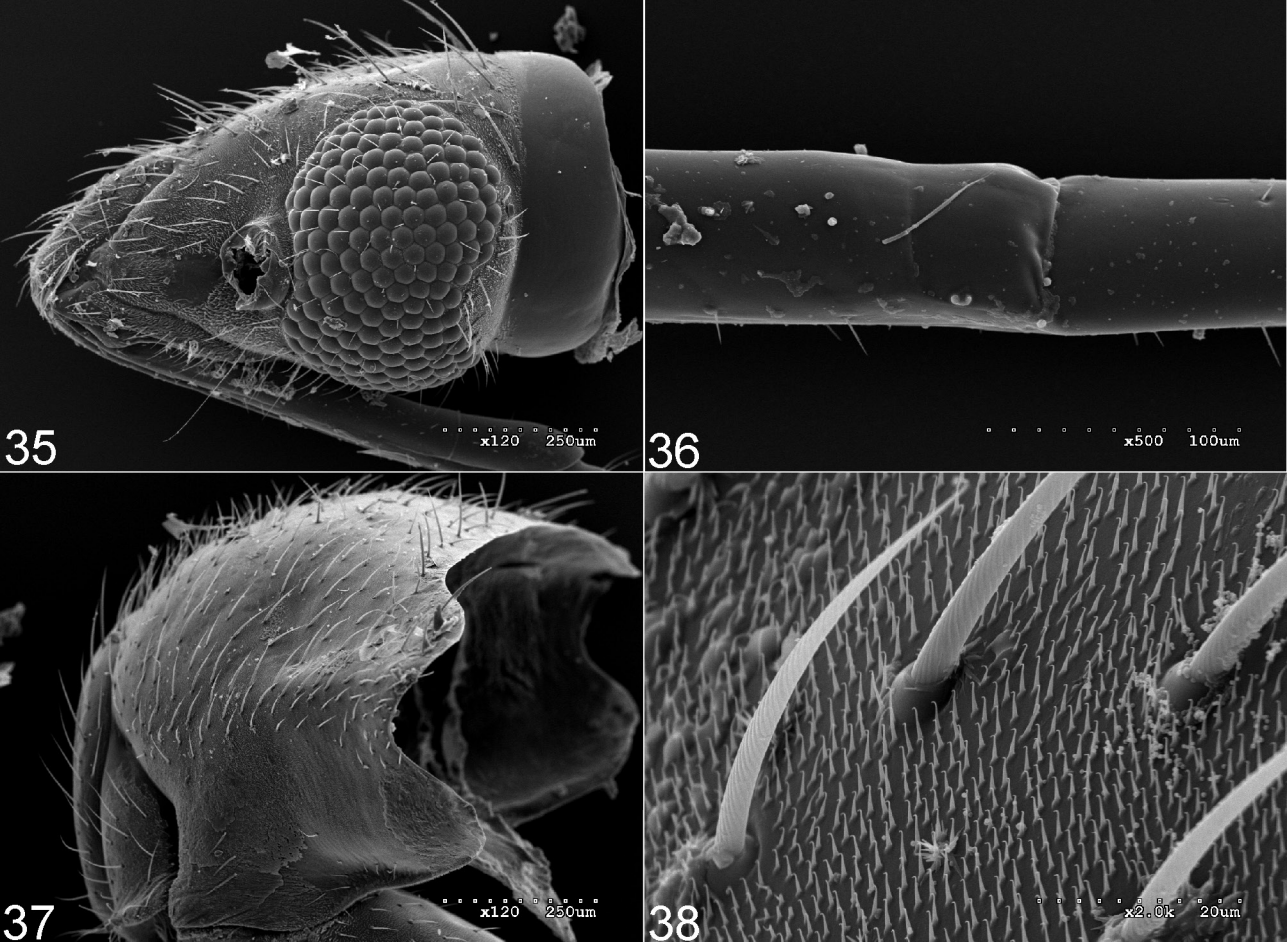
Scanning electron micrographs of *Fulviustumidipennis*. **35** Head (left lateral view) **36** Subdivision of labial segment II **37** Pronotum **38** Texture and vestiture of pronotum.

Figs 9 , 25 , 26 , 35–38

#### Diagnosis.

Eyes contiguous with pronotal collar (Figure 9); corium covered with dense setae, with yellow patches basally and apically (Figure 9). Male genitalia as in Figs 25, 26.

#### Remarks.

*Fulviustumidipennis* is most similar to *F.flavicornis* and *F.subnitens* in having a yellow patch basally and apically on the corium (Figs 5–7), but it can be distinguished by the structures of the male genitalia.

#### Description.

**Male**. **Coloration** (Figure 9). Dorsal surface varying from brown to dark brown, with yellow and orange areas. ***Head***. Dark brown; maxillary and mandibular plates sometimes slightly tinged with red; antennal segment I varying from reddish to dark brown; segment II dark brown, weakly tinged with red at basal one third, apical two thirds yellow; segments III and IV dark brownish; IV sometimes slightly yellowish apically; rostrum ranging from dark yellow to dark brown. ***Thorax***. *Pronotum*. Ranging from brown to dark brown, almost black. *Mesoscutum and scutellum*. Varying from brown to dark brown. *Thoracic pleura*. Ranging from brown, sometimes slightly tinged with red to dark brown. *Hemelytra*. Clavus and corium with yellow patch basally and apically; corium with orange patch apically, contiguous with orange patch on apex of embolium; cuneus ranging from brown to dark brown; membrane brownish, sometimes tinged with gray. *Legs*. Mostly dull yellowish, often tinged with red; coxae usually paler than remainder of leg, yellow, slightly darkened apically; femora dull yellowish, often tinged with red; tibia and tarsus yellow, rarely darkened. ***Abdomen***. Dark brown, usually tinged with yellow, rarely with red. **Structure and vestiture** (Figs 9, 35–38). Dorsal surface covered with dense, relatively long, almost decumbent setae. *Head.* Posterior margin of vertex with row of long, erect setae, present also on posterior margin of each eye; eyes slightly removed from pronotal collar; antennae covered with dense, semidecumbent setae; rostrum distinctly surpassing half of abdominal length, sometimes reaching apex. ***Abdomen***. Apical portion of abdomen flattened ventrally. **Male genitalia** (Figs 25, 26). *Left paramere* (Figure 25). Apical process and paramere body forming obtuse angle; paramere body relatively thick, left margin convex in sinistrolateral view, arcuate, right margin weakly sinuate, concave; apical process when viewed sinistrolaterally with basal two thirds cylindrical and apical third weakly tapering toward apex, ventral margin with distinct subapical spine. *Aedeagus* (Figure 26). Endosoma tumid, membranous, divided into two, large lobes apically, sclerotized portion of *ductus seminis* inside endosoma relatively long, tapering toward apex.

**Female**. Similar to male in coloration, structure, texture, and vestiture.

#### Measurements.

♀/♂: body length 4.7–4.9/4.1–5.0, width 1.5–2.0/1.3–2.0. Head. Length 0.8–0.9/0.9, width 0.6–0.6/0.6, diameter of eye in dorsal view 0.2–0.2/0.2. Antenna. Length of segment I 0.6–0.6/0.74, II 1.2–1.3/1.4, III 0.9/0.8, IV (♀): 1.2. Labium. Length of segment I 0.8–0.9/0.8, II 1.12/1.0, III 1.1/1.2, IV 0.6/0.5. Pronotum. Length 0.6–0.7/0.56–0.6, width of anterior margin 0.5–0.5/0.3–0.5, length of lateral margin 0.8–0.8/0.7, width of posterior margin 1.2-1.2/1.1-1.2.

#### Etymology.

The specific name is taken from the Latin *tumidus* (thickened) and is used to denote the distinctly thickened endosoma.

#### Biology.

Unknown.

#### Distribution.

Philippines (Mindanao).

#### Type material.

**Holotype** ♀: P.I., PHILIPPINES: Mindanao, Davao, Genitalan, 8km NW OF Mt. Apo, 690m, 17.VIII.1958; jungle clearing; light trap, H.E. Milliron; paratypes: 2 ♀♀: P.I., MINDANAO, Agusan, Los Arcos, 19–23-XI-1959; Light Trap, L. Quate & C. Yoshimoto; ♂: P.I., MINDANAO, Mis. Or., Mt. Pomalihi, 21 km W. Gingoog City, 800–1000m, 11.x.1965; H.M. Torrevillas Collector BISHOP MUSEUM; ♀: P. I. Mindanao Z. DEL SUR, 11 km NW of Milbuk, 390m, 5.VIII.1958; Logging areas in jungle; H.E. Milliron Collector (BPBM).

**Figures 39–40.**
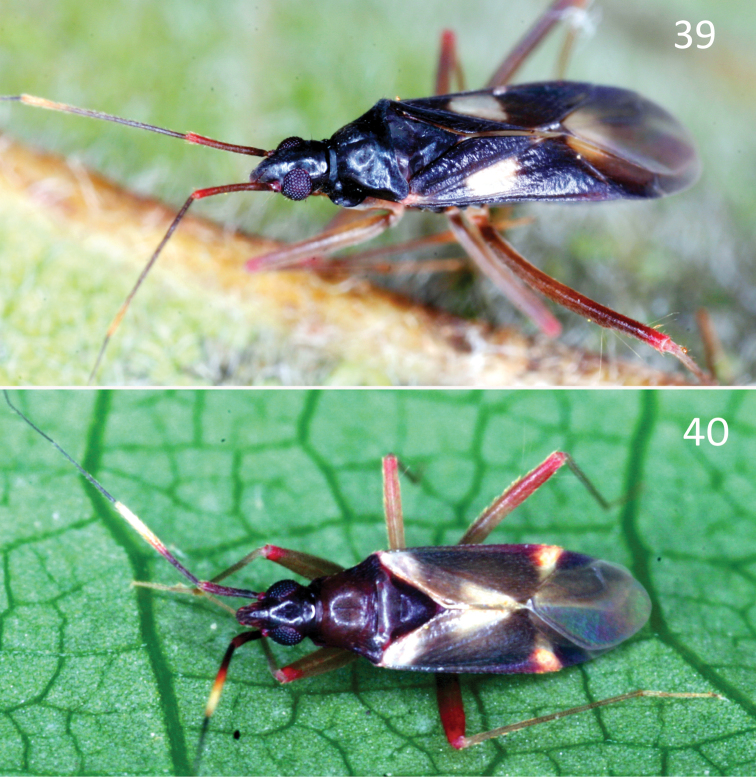
Live female adults of *Fulviusbifenestratus* (**39**) and *F.subnitens* (**40**) captured using UV light trap at Sakaerat Environmental Research Station, Thailand.

**Figures 41–48.**
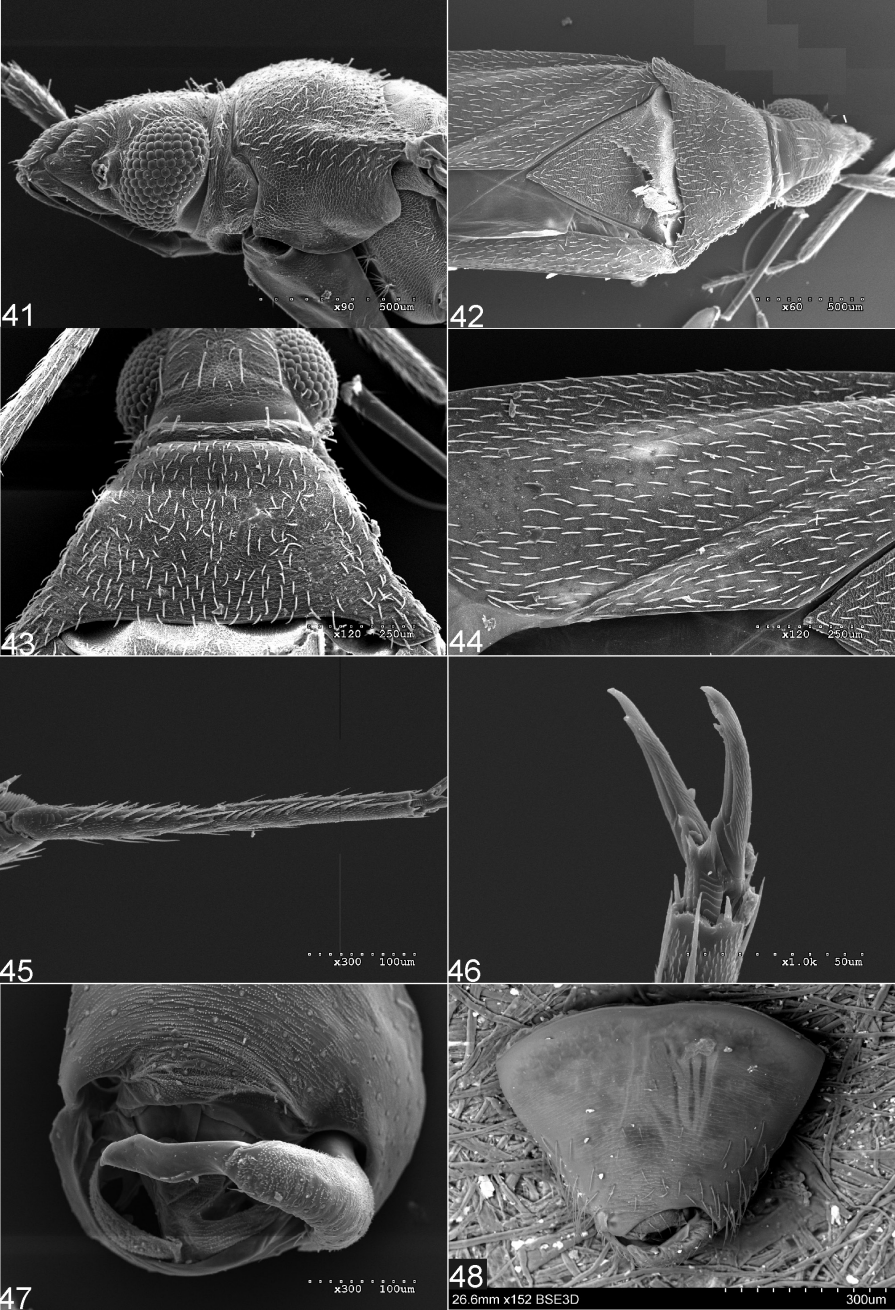
Scanning electron micrographs of *anthocoroides* group of species of the genus *Fulvius*: *F.anthocoroides* Stål (**41, 45–47**), *F.pallens* Gorczyca (**42–44**), *F.urrlichi* Sadowska-Woda & Gorczyca: (**41**). Head and pronotum (left lateral view) **42** Head, pronotum, and hemelytron (dorsal view) **43** Head and pronotum (dorsal view) **44** Vestiture of hemelytron **45** Metatarsus **46** Pretarsal claw **47, 48** Male pygophore.

**Figures 49–54.**
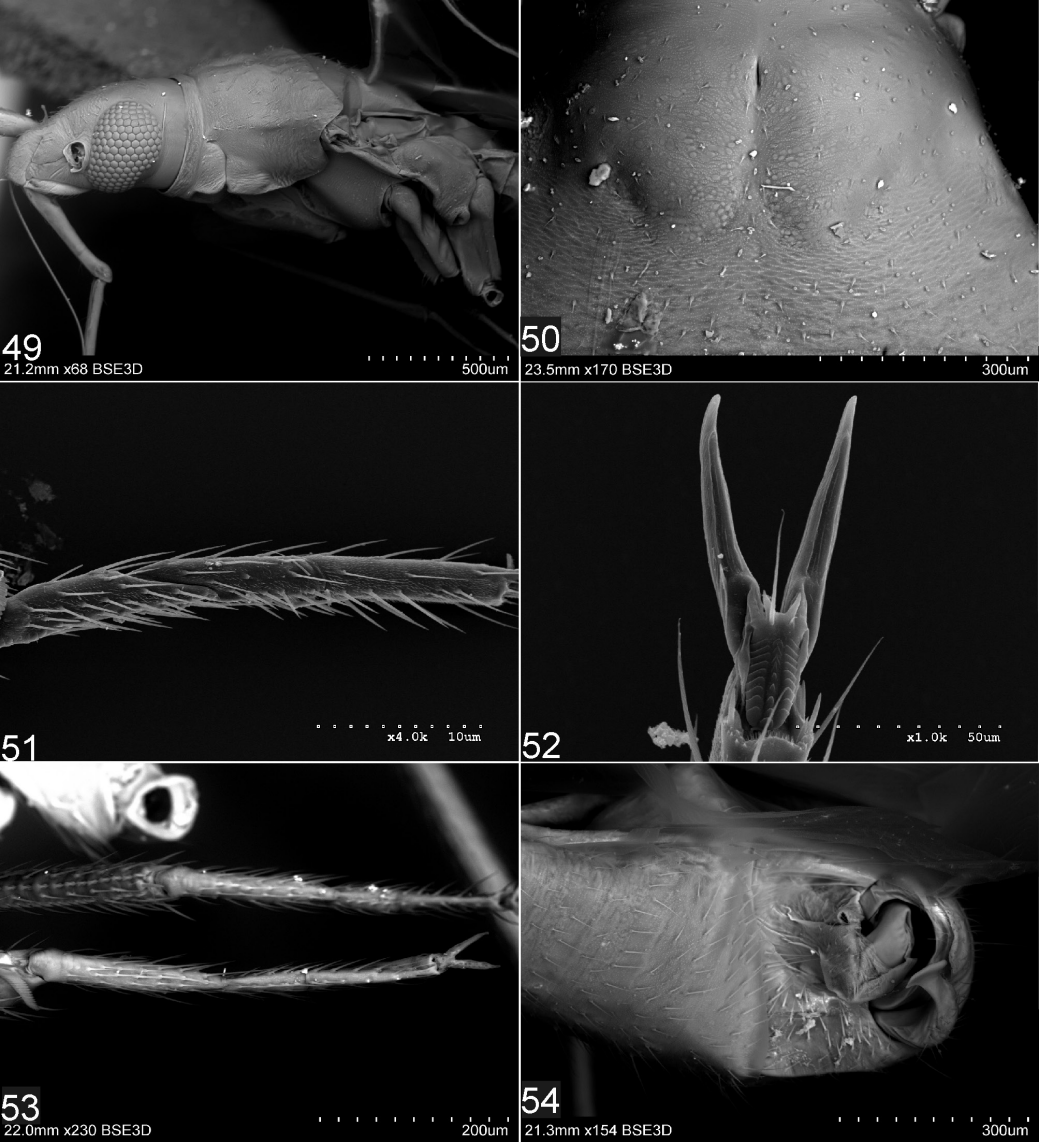
Scanning electron micrographs of *bisbistillatus* group of species of the genus *Fulvius*: *F.imbecilis* (**49–52**), *F.slateri* (**53**): **49** Head and pronotum (left lateral view) **50** Structure and vestiture of pronotum **51, 53** Metatarsus **52**. Pretarsal claw **54** Male pygophore.

## Supplementary Material

XML Treatment for Fulvius bifenestratus

XML Treatment for Fulvius bifenestratus

XML Treatment for Fulvius bimaculatus

XML Treatment for Fulvius constanti

XML Treatment for Fulvius flavicornis

XML Treatment for Fulvius henryi

XML Treatment for Fulvius subnitens

XML Treatment for Fulvius thailandicus

XML Treatment for Fulvius tumidipennis
